# The expanding syndrome of amyotrophic lateral sclerosis: a clinical and molecular odyssey

**DOI:** 10.1136/jnnp-2014-308946

**Published:** 2015-02-02

**Authors:** Martin R Turner, Michael Swash

**Affiliations:** 1 Nuffield Department of Clinical Neurosciences, University of Oxford, Oxford, UK; 2 Queen Mary University of London, London, UK; 3 University of Lisbon, Lisbon, Portugal

**Keywords:** MOTOR NEURON DISEASE, CLINICAL NEUROLOGY, GENETICS, MOLECULAR BIOLOGY, PRION

## Abstract

Recent advances in understanding amyotrophic lateral sclerosis (ALS) have delivered new questions. Disappointingly, the initial enthusiasm for transgenic mouse models of the disease has not been followed by rapid advances in therapy or prevention. Monogenic models may have inadvertently masked the true complexity of the human disease. ALS has evolved into a multisystem disorder, involving a final common pathway accessible via multiple upstream aetiological tributaries. Nonetheless, there is a common clinical core to ALS, as clear today as it was to Charcot and others. We stress the continuing relevance of clinical observations amid the increasing molecular complexity of ALS.

## Introduction

Jean-Martin Charcot (1825–1893), in his comments of 1887, underestimated the complexity of the neurodegenerative disease he named “*la sclérose amyotrophique*”:

The diagnosis as well as the anatomy and physiology of the condition amyotrophic lateral sclerosis is one of the most completely understood conditions in the realm of clinical neurology.

The history of amyotrophic lateral sclerosis (ALS) is beset by misunderstandings and false beliefs. From its 19th century beginnings, the path ahead has too often been considered straightforward, when in reality a secure information base was lacking. The enormous enthusiasm around the discovery, 20 years ago, of genetic linkage between the superoxide dismutase (*SOD1*) gene and ALS now seems unjustified. *SOD1* mutations are found in fewer than 2% of all cases, and these lack the molecular hallmark (TDP-43) common to nearly all the remainder.^1^ To ignore this group as an outlying ALS ‘phenocopy’, however, seems equally misguided. Major investments in international multicentre clinical trials appear to have been based on poorly substantiated theoretical assumptions, fuelled by a desire shared by all of us who interact with those living with ALS, to do *something* rather than do *nothing*. Unsurprisingly, they have thus far yielded disappointingly negative results.

Why then have there been so many false avenues? The answers are fundamental to extrapolating clinical observations into basic research and therapeutic trials and, eventually, into clinical practice. Debate about the historical value and ongoing reliance on animal models continues more generally in Medicine.^2^ ^3^ However, the factors leading to success or failure in the development of ideas in neuroscience, especially when applied to clinical problems, are complex. Sometimes, success follows a chance observation, albeit usually one that arises from a hypothesis-driven approach to unlock the basic pathobiology of the disorder. Here, we review the emergence of ALS as a disease, as both a clinical and now a molecular *syndrome*, and emphasise their convergence.

## The definition of a disease

As in many other diseases, Charcot's first description was formulated in the context of his insightful clinical and pathological observations with his colleague Jean Cruveilhier (1791–1874), in a climate of novel ideas that arose from several European centres. Nevertheless, the description given in Charcot's 1874 publication,^4^ in his ‘Tuesday Lectures’, and presaged in his earlier reports, set the scene for all subsequent investigations.

Charcot's genius consisted not only in describing the condition and its pathology, but also in naming it and separating it from other neurological conditions, especially those associated with muscular weakness and wasting. A name is the defining characteristic needed to facilitate universal understanding and recognition of a syndrome or, for example, in biology, of a species. An accepted nomenclature also defines and limits a syndrome. Several of Charcot's predecessors had also recognised the clinical features of ALS, for example, the Edinburgh surgeon Sir Charles Bell (1774–1842), Aran (1817–1861) who probably first recognised progressive muscular atrophy (PMA) and Cruveilhier himself. However, they failed in this essential aspect; they did not *name* the disorder. Much intellectual effort at this time was devoted to considering whether muscular wasting was due to disease of the muscle or nerve. The latter concept emerged only slowly as recognition of the importance of the separate functions of the anterior and posterior rootlets of the spinal cord developed, following the independent work of Bell in Scotland and François Magendie (1783–1855) in France. The British anatomist Jacob Augustus Lockhart Clarke (1817–1880), working privately in a room at his home, contributed to this evolving concept in relation to studies of muscular dystrophy. In 1862, with Charles Bland Radcliffe (1822–1889), Lockhart Clarke described the clinical features and the pathological findings in “An important case of paralysis and muscular atrophy, with disease of the nervous centres”, thus anticipating Charcot by 3 years, but again without naming the disorder.^5^

There was a fundamental difference between the approach taken to neurological investigation in France and Britain in the last half of the 19th century. Charcot and his followers were concerned with separating one disorder from another, for example, multiple sclerosis, epilepsy and Parkinson's disease and, especially, considering the role of psychological disorders in neurology. In Britain, there was an emphasis on understanding the *phenomena* of the disordered nervous system, rather than defining diseases per se*,* most clearly exemplified by the work of John Hughlings Jackson (1835–1911) and his pupil David Ferrier (1843–1928). This emphasis on the biology of disease, initially most frequently shared between British and German neurologists, has developed in modern times as a world-wide endeavour leading to modern neurophysiological and molecular neuroscientific studies. The resulting clinical insights led to a more robust definition of concepts such as rigidity and spasticity, delineation of particular patterns of weakness, recognition of the dystonias, and an understanding of the different types of epilepsy. Disordered mental and intellectual function was also studied as part of this late 19th century revolution, especially in subsequent years by German scientists, such as von Monakow, Liepmann and Goldstein.

## Clinical assessment in the late 19th century

For Charcot and his contemporaries, the neurological examination was far from its present form. Tendon reflexes, plantar responses, detailed testing of muscle strength designed to check characteristic patterns of weakness, modality-based sensory testing, visual field examination, pupillary reflexes and tests of muscle tone were either unknown or incompletely developed. Spasticity, as an increase in muscle tone or a disorder of stance and gait, was recognised and well described by the paediatrician and orthopaedic surgeon William John Little (1810–1894) in the context of his description of clubfoot (from which he himself suffered) and cerebral palsy.

Charcot referred in his lectures XI, XII and XIII to two patterns of motor system degeneration resulting in muscle atrophy and weakness, which he termed *protopathic* and *deuteropathic*. The former consisted of muscular atrophy and weakness associated with degeneration of the anterior cornua of the spinal cord, varying in its extent at different levels. The latter term was used to describe the combined degeneration of the anterior cornua with degeneration of the lateral columns of the cord, that is, the corticospinal tracts. However, Charcot did not correlate these pathological features with the pattern of the clinical disorder. In his Lectures, delivered at the Hôpital Salpêtriére in the period 1858–1867, he stated “the role of alterations of the nerve cells themselves had not yet been elucidated”. However, he also stated “in amyotrophic lateral sclerosis, the symmetrical lesions of the lateral columns, whence paralysis and contracture result, is the first to make its appearance; while the alteration of the anterior grey substance, with which muscular atrophy is connected, would be a consecutive phenomenon”*.* (lecture XVl), a prescient observation in relation to the modern concept that the motor cortex may be first affected in ALS.

A similar problem confronted Lockhart Clarke, himself a pioneer of histological technique in studying the nervous system, especially the spinal cord.^5^ With Radcliffe he noted that without the use of his innovative techniques: the “ordinary and inefficient method of examining the nervous centres…would have resulted in ranking the case as one of simple muscular atrophy”. He stated that the pathological changes “clearly and satisfactorily explained” the clinically evident muscular paralysis and atrophy. Lockhart Clarke's description of the pathology is remarkably complete, including atrophy of the anterior roots, particularly severe degeneration of the lateral columns in the cervical region, and atrophy of the bulbar motor nerves. Later, he also noted relative sparing of the lumbosacral cord and conus medullaris, with severe involvement of the brain stem. These important observations were acknowledged by Charcot in his 1881 Lectures. Technique, as ever in science, is everything.

Charcot noted that in ALS muscular wasting was usually more prominent in the upper limbs and rigidity was more prominent in the lower limbs. He described “fibrillary twitches of muscles” (as had Lockhart Clarke), and the limitation of the disorder to the motor system. It was left to the greatest 19th century classifier of neurological disease, William Richard Gowers (1845–1915), to suggest in his *Manual of Diseases of the Nervous System*^6^ that the several progressive disorders of the motor system could be considered as syndromic variants. This was a view with which Charcot concurred, and which led (Lord) Walter Russell Brain (1895–1966), in the 6th edition of his influential *Textbook of Neurology* published in 1962, to use the all-inclusive term Motor Neurone Disease, thus encompassing the four main clinical subtypes: ALS (essentially limb-onset disease), progressive bulbar palsy, PMA, and progressive spastic paraparesis (now termed primary lateral sclerosis, PLS). Neither Gowers nor Brain regarded these terms as indicating mutually exclusive disorders. Rather, they thought of them as describing clinical syndromes making up a single entity, the clinical features being determined by the relative distribution of pathological change in the upper motor neuronal and lower motor neuronal (UMN and LMN) systems, and its spatial distribution through the nervous system.

Charcot and Gowers both recognised that ALS appeared to begin focally and spread through the body via contiguous neuronal systems, a clinical observation that was neglected until more modern times.^7^ ^8^ Indeed, spread of the disease through the neuraxis has become a subject of contemporary re-investigation.^9^ Gowers recognised three syndromes as related. The fourth, PLS, was described by Wilhelm Heinrich Erb (1840–1921), although in the early literature it is difficult to distinguish PLS from the familial spastic paraplegias. Gowers also noted that at autopsy patients with the syndrome of PMA also showed degeneration of the corticospinal pathway, as was characteristic of ALS itself, an observation confirmed in more recent times.^10^ ^11^

**Figure 1:**
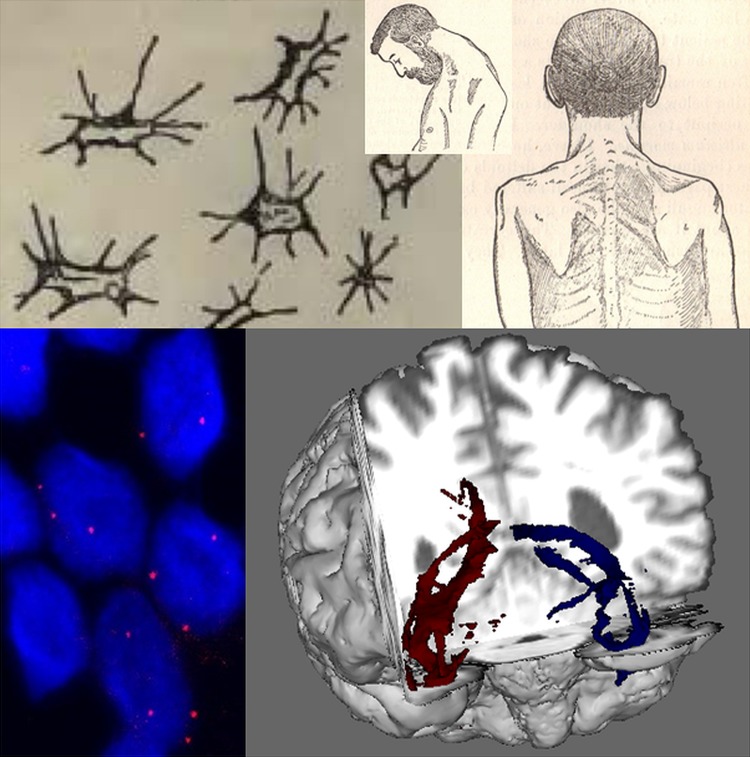
Developments in cellular and clinical probes for amyotrophic lateral sclerosis (ALS) over 130 years. Lockhart Clarke's hand-drawn atrophied anterior horn cells (top left) are contrasted with RNA foci (below, red dots) visualised within cortical neurons (nuclei, ∼5–10 µ diameter, stained blue with DAPI) differentiated from induced pluripotent stem cells derived from patient with ALS fibroblasts. Gowers’ textbook contained detailed illustrations of a classical ‘flail arm’ variant of ALS with a head drop (top middle and right), contrasted with the white matter tractography of diffusion tensor MRI (bottom right, temporal lobe projection tracts shown in a cutaway coronal plane from the front). Cortical neuron image provided courtesy of Professor Kevin Talbot, University of Oxford.

It is a central tenet of clinic-based prognostication in ALS that the rate of disease progression appears relatively fixed for an individual. The past rate of progression generally does reflect future deterioration, albeit with slightly more rapid progression in disability noted at the start and end of the disease course.^12^ Gowers was among the first to note this broad concept: “When the progress at commencement is rapid, it usually continues rapid, until the disease has attained a wide extent, although the acute local onset mentioned below may be followed by slow extension. When it begins slowly, it is usually slow throughout”*.*^13^ He also identified “a special group” which is now recognised as the ‘flail arm’ variant of ALS, and uniformly slow in its rate of progression^14^ (figure 1). It was also known to Gowers that at autopsy in ALS there are always many preserved anterior horn cells; indeed, the process of motor neuronal loss is strikingly patchy and multifocal,^7^ despite the more demarcated and sequential spread of symptoms reported by patients. Typical of the inbuilt redundancy in most human biological systems, it has been estimated that one-third of large motor neurons must be lost before there is visible atrophy.^15^

## Genetic and environmental considerations

William Osler (1849–1919) described the first cases of hereditary PMA.^16^ Gowers also recognised the occurrence of familial ALS, an observation that has assumed particular significance since the genetic revolution of modern times. Until recently, the literature on ALS was dominated by explorations of commonly assumed causative hypotheses, many derived from individual patient experience. There are many early reports implicating trauma,^17^ but unbiased hospital record linkage studies have not supported an association.^18^ Lou Gehrig, an American baseball celebrity from the 1930s, is inextricably associated with the disease in the USA. Various lifestyles, including unusual exercise regimes as in athletes,^19^ leisure-time activity,^20^ exposure to heavy work,^21^ soldiers in training,^22^ professional football players^23^ and handedness^24^ have all provided seductive associations of uncertain significance. At present, the only established risk factors for the development of sporadic ALS, apart from assumed genetic risks, are increasing age, male gender and a very modest effect of smoking. An inverse relation between dietary intake of ω-3 fatty acids and risk of ALS is the most recent observation.^25^

## ALS as an anterior brain disorder

The apparent symptomatic resistance of sensory and oculomotor neurons, along with those subserving sphincter functions, was noted in the earliest descriptions of ALS. The spinocerebellar pathway (Clarke's column), an afferent pathway that takes origin in spinal cord segments among segmental neuronal pools that are effectively largely destroyed by the disease, is a sensory pathway that is consistently affected in pathological studies of ALS. This may underlie the early symptom of impaired balance often reported by patients at diagnosis, though this has not been systematically studied. In addition, clinicians have long noted minor sensory and autonomic involvement in patients with ALS.^26^ Overlap between mild Parkinsonian features and ALS were well documented,^27^ and cross-over phenotypes have re-emerged within the spectrum of ALS linked to *C9orf72* expansions.^28^ The Guamanian ALS-Parkinson's-dementia complex represents an extreme, not least since it appears to be an acquired, non-genetic form of the disease.

The demonstration of ALS as a cerebral pathology involving regions beyond the primary motor cortex emerged first from autopsy series.^29^ Cognitive impairment and occasional psychosis were recognised among earlier descriptions of ALS (reviewed in ref. 30). The mid-1980s saw the emergence of a more subtle dysexecutive neuropsychological syndrome,^31^ followed by positron emission tomography studies providing further evidence of wide cerebral involvement in ALS.^32^ ^33^ The finding of a shared neuropathological signature of cytoplasmic ubiquitinated inclusions of the protein TDP-43 in ALS and frontotemporal dementia (FTD)^34^ is probably the most important clinicomolecular discovery in ALS research to date. Overt FTD manifests in only 10–15% of patients with ALS, typically as an early symptom,^35^ ^36^ and is strongly associated with a G_4_C_2_ hexanucleotide repeat expansion in *C9orf72.*^37^

The link between ALS and FTD represents an extension of ALS as a motor system disease to the frontal and temporal lobes, themselves parts of the brain concerned with the expression of thought, planning, personality and speech, all aspects of brain function that are strictly ‘motor’ in a wider sense. It lends support to the view that ALS pathogenesis is in some way linked to the neocortical evolutionary development of the anterior motor brain.^38^ The phrase “what wires together, dies together”^30^ focuses on an idea that there may be discrete systems whose boundaries have a role in defining the expression of degenerative processes.^39^ Thus, the long-held concept of ‘selective vulnerability’ of motor neurons has given way to a broader notion of ALS as a multisystem disease that may in part be defined by properties inherent to the motor *system* as well as the individual neuron.^40^ This can be understood as a consequence of interconnected brain networks. Motor neurodegeneration may be a process selective at that level, rather than a disorder of susceptible neuronal subtypes.^41^

## Threads of continuity between clinical and molecular taxonomies

Neurodegeneration seems to have been unmasked by the recent marked increase in the average human lifespan. Indeed, in early life, there may even be unrecognised biological advantages of mutations associated with neurodegenerative disease in the post-reproductive years.^42^ ^43^ Does sporadic ALS still exist as a valid concept,^44^ and is all ALS due to inherited factors at some level? Significantly, no major environmental risk factors have so far been discovered. At present, less than 10% of ALS in the UK population is associated with single gene mutations, the majority being expansions of the *C9orf72* hexanucleotide repeat.^45^ Next-generation sequencing offers the hope of characterising the multiple rare-variant signatures that may underlie perhaps all of the remaining cases.

Hereditary ALS and all the associated phenotypes are syndromes that can result from several different gene expressions and mutations, apparently specific to discrete protein pathways, and not all associated with ubiquitinated inclusions based on mutant TDP-43 aggregation (table 1). Mutations in *TDP-43* itself account for only a very small proportion of hereditary ALS, perhaps indicative of this gene's fundamental role in development, so that most mutations are incompatible with embryogenesis. It is axiomatic, therefore, that the common clinical phenotype, as instantly recognisable today as it was to Charcot, is an end product of upstream cellular functions that may be disrupted by a variety of mechanisms that appear disparate. These may include some or all of the excitotoxicity, neuroinflammation and mitochondrial dysfunction, for which evidence has been independently gathered over recent decades (reviewed in ref. 46). This observation is relevant to many genetically determined disorders, for example, mitochondrial disorders, limb-girdle and other muscular dystrophies, spinocerebellar degenerations, and the familial spastic paraplegia syndromes. The syndrome of ALS is therefore a single disease in the clinical sense, but a phenotype that results from a number of different, perhaps related biological abnormalities. Once initiated, or at some tipping point in a finely balanced equilibrium throughout development and early adult life, ALS appears to progress inexorably, albeit at different rates among individuals. It therefore becomes important to consider the processes underlying and modulating the common patterns of neuronal dysfunction and death in ALS, and not limit the research focus to the causative role of a monogenic mutation.

**Table 1:** Molecular clues to the core historical clinical observations in ALS, and the current gaps in knowledge

Core clinical observation	Molecular clues	Key knowledge gap
Combined UMN and LMN degeneration	Ubiquitinated neuronal inclusions found in cortical and anterior horn neuronal cell bodies^34^	Variable clinical expression of UMN versus LMN pathology, including extremes (PMA, PLS)^54^
Variable site of symptom onset	Higher proportion of bulbar-onset disease linked to *C9orf72* G_4_C_2_ expansions; and under-represented in *SOD1* mutation-associated ALS^72^	Many examples, including: Isolated bulbar variants;^73^ Reduced bulbar-onset with younger age;^74^ Lack of upper-limb onset in PLS (personal observation—MRT)
Variable age at symptom onset	*FUS* mutations linked to ALS with basophilic inclusions occur in young adults^75^	The apparent fall in incidence of ALS in those aged above age 85 years 76
Familial cases	*C9orf72* G_4_C_2_ expansions plus *SOD1*, *TDP-43*, and *FUS* mutations account for two-thirds of those with a family history of ALS or FTD^77^	Only 10% of all ALS cases carry one of these gene mutations^77^
Variable rate of disease progression	*SOD1* ‘A4V’ (dominant, rapid) versus ‘D90A’ (typically recessive, slow) mutations^78^	Typically relatively stable rates of disease progression in individual patients with ALS (familial and apparently sporadic)^12^ ^79^
Cognitive involvement	*C9orf72* G_4_C_2_ expansions strongly associated with ALS-FTD; Under-represented in *SOD1* mutations^37^	Carriers of *C9orf72* G_4_C_2_ expansions within the same pedigree may develop pure FTD instead of ALS^80^

ALS, amyotrophic lateral sclerosis; FTD, frontotemporal dementia; LMN, lower motor neuronal; PLS primary lateral sclerosis; PMA, progressive muscular atrophy; UMN, upper motor neuronal.

Studies of the *C9orf72* G_4_C_2_ hexanucleotide repeat mutation have opened a more precisely defined window into the mechanism of neuronal, and perhaps also astrocytic, degeneration in ALS which might therefore apply to nearly 10% of all cases. Repeat-associated non-ATG (RAN)-translated dipeptide products are toxic,^47^ ^48^ driving neurodegeneration by expressing abnormalities in RNA-binding proteins, perhaps through the formation of prion-like polymeric protein assemblies in the cytosol. It is suggested that a slowly accumulating toxic effect might account for the late onset and progressive course of the disease (see commentary in ref. 49). Such dipeptide products have been detected *postmortem* in a 26-year-old with learning difficulties but without dementia or motor symptoms,^50^ raising the possibility of developmental as well as degenerative influences for this expansion. However, to the clinician, the relatively abrupt onset and often rapid progression of ALS is striking, and provokes the concept of a tipping point or loss of tolerance for reasons yet to be discovered, possibly resulting from an extraneous source acting on the basis of inbuilt susceptibility. However, no such crucial external influence is currently evident.

Whether RNA or protein mishandling will prove to be pathogenic themes common to all ALS is not yet certain, and clearly it raises a fundamental question as to why such profound disturbances in basic cellular functions can be tolerated for many decades prior to the onset of symptoms, even accepting the enormous functional reserve of the nervous system. Gowers’ ‘abiotrophic doctrine’ hints that such fundamental aspects of cellular function must presumably become derailed through interaction with as yet poorly-defined age-related phenomena.^51^ ALS (and other neurodegenerative disorders) may represent a loss of the cell's capacity to safely handle proteins that have a natural tendency to aggregate, or involve a loss of quality control in protein manufacture that produces more aggregation-prone variants. Or, indeed, both processes may occur. A number of genetic mutation-driven cell stressors may be envisaged to accelerate such loss of tolerance, explaining why a *SOD1* ‘A4V’ mutation can be just as aggressive to the motor system as a pathological *C9orf72* hexanucleotide expansion. Why only the latter tends to involve cognitive impairment is not yet clear, but many scientists are focused on differential gene expression across brain regions. The process of trying to understand the biological basis for varying phenotypes within the syndrome is an ongoing challenge, and may require the use of unbiased, multifactorial, machine-learning approaches.^52^

**Figure 2:**
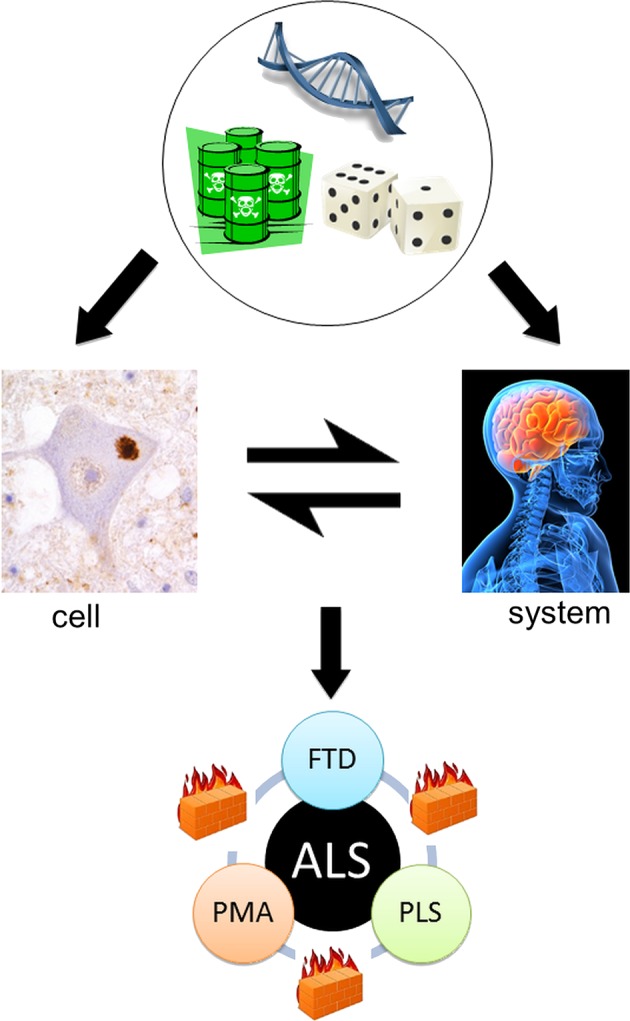
The cell versus the system in amyotrophic lateral sclerosis (after Talbot^40^). From the top: Genetic, environmental and stochastic events influence events at the cellular and also the motor *system* level. These interact with each other to result in the core ALS syndrome of mixed upper and lower motor neuron signs associated in the majority with relatively minor cognitive impairment. However, rarer pure upper motor neuron (primary lateral sclerosis, PLS), lower motor neuron (progressive muscular atrophy, PMA) and frontotemporal dementia (FTD) variants are recognised. Moreover, these are all characteristically slower in progression, possibly reflecting the relative containment of pathology due to as yet unidentified ‘firewalls’ between neuronal networks.

ALS can be recognised as a syndrome with variable clinicopathological involvement in three ‘compartments’: LMNs, UMNs and their frontotemporal connections. When the three are clinically coincident, it is associated with more rapidly progressive disease.^53^ Conversely, long survival (10–20 years) has been linked to relatively pure UMN (PLS) or LMN (PMA) involvement.^54–56^ Similarly, the median survival in ‘pure’ FTD is of the order of 10 years, suggesting a relative resistance to the spread between these three neuronal networks. Furthermore, the rate of disease progression measured according to the ALS Functional Rating Scale is largely constant over the central part of the disease course in any given patient with ALS.^12^ Whether these phenomena are a property of physical ‘wiring’, which might include a role for interneuronal circuitry^57^ or glial-neuronal interactions,^58^ is not yet clear. Much more needs to be understood concerning the potential transmission of dysfunction from cell to cell,^59^ as this might lead to a practical approach to arresting the disease. Perhaps the extremes of PLS, PMA and FTD are the natural place to start this search (figure 2).

ALS has the shortest median survival among neurodegenerative disorders. The relative infrequency of ALS may reflect the relative inaccessibility of the motor system to the protein ‘aggregopathies’ apparently common to all neurodegenerative diseases. Such a privileged status would seem likely within an evolutionary framework in which stable and reliable motor system function exerts a very large selection pressure. Once breached, however, it may be that the descending motor system (with the notable exception of oculomotor and sphincter pathways) is somehow more permissive to disease propagation, with faster progression compared to more neocortically-centred neurodegenerative processes. For example, primary FTD often shows signs of motor involvement at the end of a relatively long disease course. This is consistent with a connectivity-driven model rather than selective neuronal vulnerability, and might hinge on an initial, stochastic, regionally-defined pathological ‘seed’. This would be consistent with affected members of the same family showing different phenotypes, most recently highlighted in those carrying G_4_C_2_ expansions in *C9orf72*, where relatively pure ALS and FTD may both occur. Such reasoning has led to the suggestion that cytosolic protein aggregates in ALS behave like prions, causing dysfunction by self-replicating protein misfolding resembling the particular protein structure associated with the prion protein. Mutant SOD1 protein appears to aggregate,^60^ and RNA protein aggregates^61^ have also been reported to behave in this manner. Cell-to-cell transmission may result from pinocytosis of protein aggregates or micro-RNAs in the intercellular compartment, extruded from damaged or dead cells via exosomes.^62^ Such hypotheses would place vesicle trafficking as a strong candidate to be involved in variable progression rates observed in ALS.

Can anyone develop ALS? Given the apparent incomplete penetrance of genes associated with ALS, a more puzzling corollary is whether some individuals will *never* develop the condition, perhaps due to hitherto unexplored protective genes, limited expression of pathogenic genes across brain networks, or a less permissive neuronal milieu on a larger scale. Cohort studies, using Gompertzian statistics, have suggested that there might be a susceptible population, thus accounting for the apparently reduced incidence of the disease among the very old, while accepting that mortality at different age ranges reflects competition by different diseases.^63^

At present, we cannot identify the onset of the disease other than by the patient's observations of the onset of focal weakness and wasting, or mental change, but these onset features are vague in their timing, and must represent much earlier changes that exist subclinically for many months or even years.^64^ The study of presymptomatic individuals carrying highly-penetrant ALS gene mutations is an important emerging initiative.^65^ Continuing discussion as to whether ALS begins in the motor cortex, as suggested by the finding of increased excitability (decreased inhibition) of the motor cortex prior to the onset of symptoms,^66^ or simultaneously in various sites in the motor system^67^ is consistent with concepts of spreading pathology in other neurodegenerative diseases, such as Parkinson's.^68^ ^69^ Consistent patterns of preferential muscle involvement in ALS, such as the lateral hand muscle wasting known as the ‘split hand’, have been linked to cortical representations associated with the development of the opposable thumb.^70^ The fasciculation so characteristic of ALS is itself evidence of the increased excitability at a lower motor neuronal level.^71^

## Concluding remarks

Careful clinical observation has been pivotal to the current understanding of ALS. The first essential in the story was to give the disorder a name based on the core clinical features that remain instantly recognisable today despite the new taxonomies based on single gene defects or the predominant cellular inclusion. Gowers was a ‘lumper’, not a ‘splitter’, and Lord Brain followed with the term MND.

Let us keep looking in spite of everything. Let us keep searching. It is indeed the best method of finding, and perhaps thanks to our efforts, the verdict we will give such a patient tomorrow will not be the same we must give this patient today.

Charcot's call to activity has not lost its urgency over 125 years later. Until more is understood about the *common* underlying pathophysiology, along with a greater appreciation of the at-risk population for the apparently sporadic disorder, history may judge current therapeutic trials in ALS to have been misguided. Current research efforts aimed at understanding the basic pathobiology of the disease are proposed to lead to putative therapies, which should be testable against outcomes in clinical trials. Clinical trials have also proved to be of great benefit in terms of understanding the natural history of ALS, in improving patient management, and in fostering a scientific environment from which quantum leaps in understanding will emerge in time, perhaps serendipitously.

Charcot's classical patient-centred approach has moved from the Lecture Theatre to the Laboratory, where it must now both inform and reflect research activity. Fibroblast-derived, induced pluripotent stem cells hold promise of *individualised* models of disease. This is a reality that could not have been predicted, but which holds genuine hope for delivering the long-awaited breakthrough in therapy or, better still, prevention. However, expansion in the clinical syndrome of ALS has occurred *despite*, rather than as a result of, new molecular insights, and will remain fundamental to the correct interpretation of the latter.

## References

[1] Mackenzie IR, Bigio EH, Ince PG, et al Pathological TDP-43 distinguishes sporadic amyotrophic lateral sclerosis from amyotrophic lateral sclerosis with SOD1 mutations. Ann Neurol 2007;61:427–34. 17469116 10.1002/ana.21147

[2] Pound P, Ebrahim S, Sandercock P, et al Where is the evidence that animal research benefits humans? BMJ 2004;328:514–17. 14988196 10.1136/bmj.328.7438.514 PMC351856

[3] Pound P, Bracken MB Is animal research sufficiently evidence based to be a cornerstone of biomedical research? BMJ 2014;348:g3387. 24879816 10.1136/bmj.g3387

[4] Charcot J-M Amyotrophies spinales deuteropathiques sclérose latérale amyotrophique & Sclérose latérale amyotrophique. Bureaux du Progrès Médical 1874;2 (Oeuvres Complétes):234–66.

[5] Turner MR, Swash M, Ebers GC Lockhart Clarke's contribution to the description of amyotrophic lateral sclerosis. Brain 2010;133:3470–9. 20576696 10.1093/brain/awq097 PMC3182545

[6] Gowers WR A Manual of diseases of the nervous system. London: J & A Churchill, 1886.

[7] Swash M, Leader M, Brown A, et al Focal loss of anterior horn cells in the cervical cord in motor neuron disease. Brain 1986;109 (Pt 5):939–52. 3779374 10.1093/brain/109.5.939

[8] Swash M, Ingram D Preclinical and subclinical events in motor neuron disease. J Neurol Neurosurg Psychiatry 1988;51:165–8. 3346681 10.1136/jnnp.51.2.165 PMC1031524

[9] Ravits JM, La Spada AR ALS motor phenotype heterogeneity, focality, and spread: deconstructing motor neuron degeneration. Neurology 2009;73:805–11. 19738176 10.1212/WNL.0b013e3181b6bbbd PMC2739608

[10] Brownell B, Oppenheimer DR, Hughes JT The central nervous system in motor neurone disease. J Neurol Neurosurg Psychiatry 1970;33:338–57. 5431724 10.1136/jnnp.33.3.338 PMC493478

[11] Ince PG, Evans J, Knopp M, et al Corticospinal tract degeneration in the progressive muscular atrophy variant of ALS. Neurology 2003;60:1252–8. 12707426 10.1212/01.wnl.0000058901.75728.4e

[12] Gordon PH, Cheng B, Salachas F, et al Progression in ALS is not linear but is curvilinear. J Neurol 2010;257:1713–17. 20532545 10.1007/s00415-010-5609-1

[13] Gowers WR Chronic Spinal Muscular Atrophy. A Manual of Diseases of the Nervous System. 11893. p. 483.

[14] Hu MT, Ellis CM, Al Chalabi A, et al Flail arm syndrome: a distinctive variant of amyotrophic lateral sclerosis. J Neurol Neurosurg Psychiatry 1998;65:950–1. 9854987 10.1136/jnnp.65.6.950 PMC2170397

[15] Wohlfart G Collateral regeneration from residual motor nerve fibers in amyotrophic lateral sclerosis. Neurology 1957;7:124–34. 13400219 10.1212/wnl.7.2.124

[16] Osler W On heredity in progressive muscular atrophy as illustrated in the Farr family of Vermont. Arch Med 1880;4:316–20.

[17] Erb WH Zur Lehre von den Unfallerkrankungen des Rückenmarkes, über Poliomyelitis anterior chronica nach Trauma. Dtsch Nervenheilk 1897;11:122–42.

[18] Turner MR, Abisgold J, Yeates DG, et al Head and other physical trauma requiring hospitalisation is not a significant risk factor in the development of ALS. J Neurol Sci 2010;288:45–8. 19878957 10.1016/j.jns.2009.10.010

[19] Scarmeas N, Shih T, Stern Y, et al Premorbid weight, body mass, and varsity athletics in ALS. Neurology 2002;59:773–5. 12221178 10.1212/wnl.59.5.773

[20] Huisman MH, Seelen M, de Jong SW, et al Lifetime physical activity and the risk of amyotrophic lateral sclerosis. J Neurol Neurosurg Psychiatry 2013;84:976–81. 23418211 10.1136/jnnp-2012-304724

[21] Beghi E, Logroscino G, Chio A, et al Amyotrophic lateral sclerosis, physical exercise, trauma and sports: results of a population-based pilot case-control study. Amyotroph Lateral Scler 2010;11:289–92. 20433412 10.3109/17482960903384283 PMC3513269

[22] Weisskopf MG, O'Reilly EJ, McCullough ML, et al Prospective study of military service and mortality from ALS. Neurology 2005;64:32–7. 15642900 10.1212/01.WNL.0000148649.17706.D9

[23] Chio A, Benzi G, Dossena M, et al Severely increased risk of amyotrophic lateral sclerosis among Italian professional football players. Brain 2005;128 (Pt 3):472–6. 15634730 10.1093/brain/awh373

[24] Turner MR, Wicks P, Brownstein CA, et al Concordance between site of onset and limb dominance in amyotrophic lateral sclerosis. J Neurol Neurosurg Psychiatry 2010;82:853–4. 20562391 10.1136/jnnp.2010.208413

[25] Fitzgerald KC, O'Reilly EJ, Falcone GJ, et al Dietary omega-3 polyunsaturated fatty acid intake and risk for amyotrophic lateral sclerosis. JAMA Neurol 2014;71:1102–10. 25023276 10.1001/jamaneurol.2014.1214 PMC4160351

[26] Wechsler IS, Brock S, Weil A Amyotrophic lateral sclerosis with objective and subjective (neuritic) sensory disturbances. Arch Neurol Psychiatry 1929;21:299–310.

[27] Hudson AJ Amyotrophic lateral sclerosis and its association with dementia, parkinsonism and other neurological disorders: a review. Brain 1981;104:217–47. 7016254 10.1093/brain/104.2.217

[28] Origone P, Verdiani S, Ciotti P, et al Enlarging the clinical spectrum associated with C9orf 72 repeat expansions: findings in an Italian cohort of patients with parkinsonian syndromes and relevance for genetic counselling. Amyotroph Lateral Scler Frontotemporal Degener 2013;14:479–80. 23509957 10.3109/21678421.2013.774020

[29] Smith MC Nerve fibre degeneration in the brain in amyotrophic lateral sclerosis. J Neurol Neurosurg Psychiatry 1960;23:269–82. 21610893 10.1136/jnnp.23.4.269 PMC497425

[30] Bak TH, Chandran S What wires together dies together: verbs, actions and neurodegeneration in motor neuron disease. Cortex 2012;48:936–44. 21924711 10.1016/j.cortex.2011.07.008

[31] Gallassi R, Montagna P, Ciardulli C, et al Cognitive impairment in motor neuron disease. Acta Neurol Scand 1985;71:480–4. 4024859 10.1111/j.1600-0404.1985.tb03231.x

[32] Dalakas MC, Hatazawa J, Brooks RA, et al Lowered cerebral glucose utilization in amyotrophic lateral sclerosis. Ann Neurol 1987;22:580–6. 3501273 10.1002/ana.410220504

[33] Ludolph AC, Langen KJ, Regard M, et al Frontal lobe function in amyotrophic lateral sclerosis: a neuropsychologic and positron emission tomography study. Acta Neurol Scand 1992;85:81–9. 1574993 10.1111/j.1600-0404.1992.tb04003.x

[34] Neumann M, Sampathu DM, Kwong LK, et al Ubiquitinated TDP-43 in frontotemporal lobar degeneration and amyotrophic lateral sclerosis. Science 2006;314:130–3. 17023659 10.1126/science.1134108

[35] Phukan J, Elamin M, Bede P, et al The syndrome of cognitive impairment in amyotrophic lateral sclerosis: a population-based study. J Neurol Neurosurg Psychiatry 2012;83:102–8. 21836033 10.1136/jnnp-2011-300188

[36] Elamin M, Bede P, Byrne S, et al Cognitive changes predict functional decline in ALS: A population-based longitudinal study. Neurology 2013;80:1590–7. 23553481 10.1212/WNL.0b013e31828f18ac

[37] Byrne S, Elamin M, Bede P, et al Cognitive and clinical characteristics of patients with amyotrophic lateral sclerosis carrying a C9orf72 repeat expansion: a population-based cohort study. Lancet Neurol 2012;11:232–40. 22305801 10.1016/S1474-4422(12)70014-5 PMC3315021

[38] Eisen A, Turner MR, Lemon R Tools and talk: an evolutionary perspective on the functional deficits associated with amyotrophic lateral sclerosis. Muscle Nerve 2014;49:469–77. 24273101 10.1002/mus.24132

[39] Zhou J, Gennatas ED, Kramer JH, et al Predicting regional neurodegeneration from the healthy brain functional connectome. Neuron 2012;73:1216–27. 22445348 10.1016/j.neuron.2012.03.004 PMC3361461

[40] Talbot K Amyotrophic lateral sclerosis: cell vulnerability or system vulnerability? J Anat 2014;224:45–51. 24010870 10.1111/joa.12107 PMC3867886

[41] Eisen A, Turner MR Does variation in neurodegenerative disease susceptibility and phenotype reflect cerebral differences at the network level? Amyotrophic Lateral Scler Frontotemporal Degener 2013;14:487–93. 10.3109/21678421.2013.812660 23879681

[42] Turner M, Goldacre R, Goldacre M Reduced cancer incidence in Huntington's disease: record linkage study clue to an evolutionary trade-off? Clin Genet 2013;83:588–90. 23017147 10.1111/cge.12010

[43] Raichlen DA, Alexander GE Exercise, APOE genotype, and the evolution of the human lifespan. Trends Neurosci 2014;37:247–55. 24690272 10.1016/j.tins.2014.03.001 PMC4066890

[44] Al-Chalabi A, Lewis CM Modelling the effects of penetrance and family size on rates of sporadic and familial disease. Hum Hered 2011;71:281–8. 21846995 10.1159/000330167

[45] Andersen PM, Al-Chalabi A Clinical genetics of amyotrophic lateral sclerosis: what do we really know? Nat Rev Neurol 2011;7:603–15. 21989245 10.1038/nrneurol.2011.150

[46] Turner MR, Bowser R, Bruijn L, et al Mechanisms, models and biomarkers in amyotrophic lateral sclerosis. Amyotrophic Lateral Scler Frontotemporal Degener 2013;14 (Suppl 1):19–32. 10.3109/21678421.2013.778554 PMC4284067 23678877

[47] Mizielinska S, Gronke S, Niccoli T, et al C9orf72 repeat expansions cause neurodegeneration in Drosophila through arginine-rich proteins. Science 2014;345:1192–4. 25103406 10.1126/science.1256800 PMC4944841

[48] Kwon I, Xiang S, Kato M, et al Poly-dipeptides encoded by the C9orf72 repeats bind nucleoli, impede RNA biogenesis, and kill cells. Science 2014;345:1139–45. 25081482 10.1126/science.1254917 PMC4459787

[49] West JP III, Gitler AD Cell Biology. Clogging information flow in ALS. Science 2014;345:1118–19. 25190778 10.1126/science.1259461

[50] Proudfoot M, Gutowski NJ, Edbauer D, et al Early dipeptide repeat pathology in a frontotemporal dementia kindred with C9ORF72 mutation and intellectual disability. Acta Neuropathol 2014;127:451–8. 24445903 10.1007/s00401-014-1245-7

[51] Gowers WR A lecture on abiotrophy. Lancet 1902;i:1003–7.

[52] Blain CR, Brunton S, Williams VC, et al Differential corticospinal tract degeneration in homozygous ‘D90A’ SOD-1 ALS and sporadic ALS. J Neurol Neurosurg Psychiatry 2011;82:843–9. 21515558 10.1136/jnnp.2010.236018 PMC3134064

[53] Elamin M, Phukan J, Bede P, et al Executive dysfunction is a negative prognostic indicator in patients with ALS without dementia. Neurology 2011;76:1263–9. 21464431 10.1212/WNL.0b013e318214359f

[54] Swank RL, Putnam TJ Amyotrophic lateral sclerosis and related conditions: a clinical analysis. Arch Neurol Psychiat 1943;49:151–77.

[55] Turner MR, Parton MJ, Shaw CE, et al Prolonged survival in motor neuron disease: a descriptive study of the King's database 1990–2002. J Neurol Neurosurg Psychiatry 2003;74:995–7. 12810805 10.1136/jnnp.74.7.995 PMC1738535

[56] Chio A, Calvo A, Moglia C, et al Phenotypic heterogeneity of amyotrophic lateral sclerosis: a population based study. J Neurol Neurosurg Psychiatry 2011;82:740–6. 21402743 10.1136/jnnp.2010.235952

[57] Turner MR, Kiernan MC Does interneuronal dysfunction contribute to neurodegeneration in amyotrophic lateral sclerosis? Amyotroph Lateral Scler 2012;13:245–50. 22424125 10.3109/17482968.2011.636050

[58] Philips T, Robberecht W Neuroinflammation in amyotrophic lateral sclerosis: role of glial activation in motor neuron disease. Lancet Neurol 2011;10:253–63. 21349440 10.1016/S1474-4422(11)70015-1

[59] Swash M How does ALS spread between neurones in the CNS? J Neurol Neurosurg Psychiatry 2013;84:116–17. 23033352 10.1136/jnnp-2012-303992

[60] Munch C, O'Brien J, Bertolotti A Prion-like propagation of mutant superoxide dismutase-1 misfolding in neuronal cells. Proc Natl Acad Sci USA 2011;108:3548–53. 21321227 10.1073/pnas.1017275108 PMC3048161

[61] Kim HJ, Kim NC, Wang YD, et al Mutations in prion-like domains in hnRNPA2B1 and hnRNPA1 cause multisystem proteinopathy and ALS. Nature 2013;495:467–73. 23455423 10.1038/nature11922 PMC3756911

[62] Prusiner SB Cell biology. A unifying role for prions in neurodegenerative diseases. Science 2012;336:1511–13. 22723400 10.1126/science.1222951 PMC3942086

[63] Neilson S, Robinson I, Alperovitch A Rising amyotrophic lateral sclerosis mortality in France 1968–1990: increased life expectancy and inter-disease competition as an explanation. Journal Neurol 1994;241:448–55. 10.1007/BF00900964 7931447

[64] Eisen A, Kiernan M, Mitsumoto H, et al Amyotrophic lateral sclerosis: a long preclinical period? J Neurol Neurosurg Psychiatry 2014;85:1232–8. 24648037 10.1136/jnnp-2013-307135

[65] Benatar M, Wuu J Presymptomatic studies in ALS: rationale, challenges, and approach. Neurology 2012;79:1732–9. 23071166 10.1212/WNL.0b013e31826e9b1d PMC3468777

[66] Vucic S, Nicholson GA, Kiernan MC Cortical hyperexcitability may precede the onset of familial amyotrophic lateral sclerosis. Brain 2008;131 (Pt 6):1540–50. 18469020 10.1093/brain/awn071

[67] Swash M Amyotrophic lateral sclerosis: a phylogenetic disease of the corticomotoneuron? Comments on the hypothesis. Muscle Nerve 1992;15:226–8. 1549145 10.1002/mus.880150217

[68] Braak H, Del Tredici K, Rub U, et al Staging of brain pathology related to sporadic Parkinson's disease. Neurobiol Aging 2003;24:197–211. 12498954 10.1016/s0197-4580(02)00065-9

[69] Brettschneider J, Del Tredici K, Toledo JB, et al Stages of pTDP-43 pathology in amyotrophic lateral sclerosis. Ann Neurol 2013;74:20–38. 23686809 10.1002/ana.23937 PMC3785076

[70] Eisen A, Kuwabara S The split hand syndrome in amyotrophic lateral sclerosis. J Neurol Neurosurg Psychiatry 2012;83:399–403. 22100761 10.1136/jnnp-2011-301456

[71] de Carvalho M, Swash M Fasciculation potentials and earliest changes in motor unit physiology in ALS. J Neurol Neurosurg Psychiatry 2013;84:963–8. 23418210 10.1136/jnnp-2012-304545

[72] Millecamps S, Boillee S, Le Ber I, et al Phenotype difference between ALS patients with expanded repeats in C9ORF72 and patients with mutations in other ALS-related genes. J Med Genet 2012;49:258–63. 22499346 10.1136/jmedgenet-2011-100699

[73] Burrell JR, Vucic S, Kiernan MC Isolated bulbar phenotype of amyotrophic lateral sclerosis. Amyotroph Lateral Scler 2011;12:283–9. 21702735 10.3109/17482968.2011.551940

[74] Turner MR, Barnwell J, Al-Chalabi A, et al Young-onset amyotrophic lateral sclerosis: historical and other observations. Brain 2012;135 (Pt 9):2883–91. 22661746 10.1093/brain/aws144

[75] Baumer D, Hilton D, Paine SM, et al Juvenile ALS with basophilic inclusions is a FUS proteinopathy with FUS mutations. Neurology 2010;75:611–18. 20668261 10.1212/WNL.0b013e3181ed9cde PMC2931770

[76] Eisen A, Krieger C Epidemiological considerations. Amyotrophic lateral sclerosis: Cambridge University Press, 1998:1–4.

[77] Leblond CS, Kaneb HM, Dion PA, et al Dissection of genetic factors associated with amyotrophic lateral sclerosis. Exp Neurol 2014. 10.1016/j.expneurol.2014.04.013 24780888

[78] Andersen PM, Nilsson P, Keranen ML, et al Phenotypic heterogeneity in motor neuron disease patients with CuZn-superoxide dismutase mutations in Scandinavia. Brain 1997;120 (Pt 10):1723–37. 9365366 10.1093/brain/120.10.1723

[79] Kimura F, Fujimura C, Ishida S, et al Progression rate of ALSFRS-R at time of diagnosis predicts survival time in ALS. Neurology 2006;66:265–7. 16434671 10.1212/01.wnl.0000194316.91908.8a

[80] Majounie E, Renton AE, Mok K, et al Frequency of the C9orf72 hexanucleotide repeat expansion in patients with amyotrophic lateral sclerosis and frontotemporal dementia: a cross-sectional study. Lancet Neurol 2012;11:323–30. 22406228 10.1016/S1474-4422(12)70043-1 PMC3322422

